# Prediction of speed of sound of deep eutectic solvents using artificial neural network coupled with group contribution approach

**DOI:** 10.1038/s41598-025-14094-w

**Published:** 2025-08-10

**Authors:** Ayat Hussein Adhab, Morug Salih Mahdi, Hardik Doshi, Anupam Yadav, R. Manjunatha, Sushil Kumar, Debasish Shit, Gargi Sangwan, Aseel Salah Mansoor, Usama Kadem Radi, Nasr Saadoun Abd

**Affiliations:** 1 Department of Pharmacy, Al-Zahrawi University College, Karbala, Iraq; 2 College of MLT, Ahl Al Bayt University, Karbala, Iraq; 3 Department of Computer Engineering, Faculty of Engineering and Technology, Marwadi University Research Center, Marwadi University, Rajkot, Gujarat 360003 India; 4 Department of Computer Engineering and Application, GLA University, Mathura, 281406 India; 5 Department of Data Analytics and Mathematical Sciences, School of Sciences, JAIN (Deemed to be University), Bangalore, Karnataka India; 6 Department of Computer Application, Chandigarh Engineering College, Chandigarh Group of Colleges-Jhanjeri, Mohali, Punjab 140307 India; 7 Centre for Research Impact and Outcome, Chitkara University Institute of Engineering and Technology, Chitkara University, Rajpura, Punjab 140401 India; 8 Chitkara Centre for Research and Development, Chitkara University, Baddi, Himachal Pradesh 174103 India; 9 Gilgamesh Ahliya University, Baghdad, Iraq; 10 Collage of Pharmacy, National University of Science and Technology, Dhi Qar, 64001 Iraq; 11 Medical Technical College, Al-Farahidi University, Baghdad, Iraq

**Keywords:** Artificial intelligence, Neural network, Speed of sound, Deep eutectic, Group contribution, Machine learning, CatBoost, Chemical engineering, Chemical engineering

## Abstract

Predicting the physiochemical properties of deep eutectic solvents (DESs) is crucial for designing new solvents. Heat capacity and speed of sound are important thermodynamic properties in chemical processes. However, experimental data on the speed of sound in DESs is limited. Consequently, a thermodynamic model is needed to estimate the speed of sound in DESs over a wide range of pressures and temperatures. A key challenge in these models is accurately estimating the ideal gas heat capacity. Since the ideal gas heat capacity of DESs is often unavailable, a machine learning (ML) approach, using artificial neural networks (ANNs) coupled with a Group Contribution (GC) method, is a promising technique. The GC approach will be used to estimate critical temperature, volume, and acentric factor of DESs, which can then be input into the ANN model to predict the speed of sound. The results show that using a combination of a GC method and ANNs or CatBoost ML provides a highly accurate prediction of the speed of sound in DESs. Input parameters to the ANN + GC include temperature, acentric factor, molecular weight, and critical volume. The absolute relative deviation (ARD%) and R^2^ values of correlated speed of sound for the ANN + GC model have been obtained 0.032% and 0.998, respectively. The ARD% for both the ANN + GC and ML + GC approaches was substantially lower than that of the correlation-based models. Furthermore, cumulative frequency diagrams and the leverage approach were implemented to validate the quality and reliability of the proposed model. The leverage analysis confirmed the accuracy of the data used and the high reliability of the ANN + GC model for estimating the speed of sound in DESs. This analysis indicates that the ANN + GC and ML + GC methods can effectively estimate the speed of sound in DESs based on molecular structure. Therefore, these approaches offer a promising tool for predicting the speed of sound of newly designed DESs when experimental data is unavailable.

## Introduction

Deep eutectic solvents (DESs) are mixtures of a hydrogen bond donor (HBD) and a hydrogen bond acceptor (HBA), which combine to form a solvent with a melting point lower than that of either individual component. Their advantageous properties, including low toxicity and low vapor pressure, have led to significant interest in DESs across a variety of fields^1^. These types of solvents are considered an alternative to traditional organic solvents. Similar to ionic liquid (IL), DES physicochemical properties can be tuned by the suitable combination of HBD–HBA compounds. It must be noted that ILs and DESs are different due to the nature of starting materials and the methods for their formation. When considering starting materials and formation methods, ILs are mixtures of cations and anions, requiring synthesis with reagents and solvents. In contrast, DESs consist of HBAs and HBDs, and they can be prepared using single components through a simple heat treatment^2^. DESs are widely used in pharmacology^3^, gas-capturing processes (especially CO_2_ capture)^4^, extraction operations^5–7^, and water treatment^8^. The low vapor pressure (almost negligible vapor pressure) of DESs is one of their main properties^9^. In industry, environmentally friendlier and greener solvent alternatives for the manufacturing of their products are required^10^. Bowen et al. have demonstrated the utility of DESs in protein extraction and purification^10^ highlighting their potential as a promising, alternative solvent for protein extraction from diverse raw biomass sources. Furthermore, recent research^11^ has focused on designing green, highly efficient, and recyclable DESs for separating EVA films from end-of-life (EOL) photovoltaic (PV) modules. The inherent high-temperature stability and acidic nature of DESs can effectively facilitate the separation of EVA film layers within these modules^11^. Jahanbakhsh-Bonab et al. used molecular dynamics (MD) simulations to examine the physicochemical and structural characteristics of novel DESs^12^. Research indicates that methyl-β-cyclodextrin (MBCD)-based DESs can provide predictive insights for their applications in extraction processes. Furthermore, studies have explored the structural and physicochemical properties of chiral DESs, composed of racemic mixtures of menthol with acetic acid, menthol with lauric acid, and menthol with pyruvic acid, specifically for enantioselective extraction processes^13^. Jahanbakhsh-Bonab et al. utilized the MD simulations to examine the structural and dynamical properties of DESs-based boron nitride nanotube (BNNT) nanofluid^14^. The impact of nanotube diameter on the physicochemical parameters of DES-based systems has been investigated. Results indicate that adding Boron Nitride Nanotubes (BNNTs) to DESs increases viscosity due to a reduction in the diffusion coefficient of the DES components. Understanding the structure of DES-based nanofluids is crucial for comprehending the properties of these novel solvents in chemical processes. Moreover, MD simulations have been employed to examine the effects of external electric fields (EEFs) on the structural and transport properties of DESs composed of a 2:1 molar ratio of glycerol (Gly) and choline chloride (ChCl)^15^. They calculated several key physicochemical properties of Gly/ChCl DESs in the absence of external electric fields (EEFs), including viscosity, self-diffusion coefficient, isothermal compressibility, and density. They also employed the radial distribution function (RDF), coordination number, and number of hydrogen bonds to analyze the arrangement of the DES components. Their findings indicated that the correlation of movement between glycerol (Gly) and chloride (Cl) ions decreases as the strength of the EEF increases. Furthermore, in recent years, MD simulations have been increasingly used to investigate the performance of DESs in separating acid gases from natural gas^16^. The performance of DESs was compared with that of methyl diethanolamine (MDEA) system. The results show that, the diffusion coefficient of H_2_S and CO_2_ follow the trends Nano-DES < DES-MDEA < DES < MDEA < aqueous MDEA, and DES < DES-MDEA < MDEA < Nano-DES < aqueous MDEA in the liquid phase. Also, the performance of an amine-based DES composed of a 6:1 molar ratio of methyl diethanolamine (MDEA) and choline chloride (ChCl) for the natural gas (NG) sweetening process was investigated using MD simulations^17^. The effect of pressure on the performance of amine-based DESs for natural gas sweetening has been examined. The results suggest that DESs based on N-methyldiethanolamine (MDEA) can compete with conventional amine solvents in natural gas sweetening processes. P. Jahanbakhsh-Bonab et al. simulated the absorption capability of DESs based on MDEA using MD simulations^18^. Also, the effect of temperature on absorption capability was studied. The thermophysical properties of monoethanolamine (MEA)-based DESs for H_2_S extraction from biogas were investigated^19^. The impact of pressure on the performance of amine-based DESs for the extraction of H_2_S and CO_2_ was studied. Esfahani et al. used choline chloride-based DESs for the extraction of 1-butanol or 2-butanol from azeotropic n-heptane + butanol mixtures. This shows a difference in application and the type of DES used^7^. In summary, the prediction of thermodynamic properties of DESs plays an important role in chemical processes. The first-order derivative thermodynamic properties such as density, and second-order derivative thermodynamic properties such as heat capacity and speed of sound of the system, must be estimated in the pre-design of a new chemical process. Predictive models are more cost-effective than experimentally measuring the properties of a large number of potential designs (DESs) for a chemical process. M. Taherzadeh et al. proposed a correlation-based model to correlate/predict the heat capacity of 28 DESs over the wide temperature range from 278 to 363 K^20^. The proposed correlation was developed based on molecular weight, temperature, acentric factor, and critical pressure. The absolute average relative deviation (AARD%) of the model for all of the studied data points was 4.7%. Leron and Li created a model specifically for estimating the heat capacity of DES^21^. Naser et al. developed a model for the specific heat capacity calculation of 15 DESs^22^. Zhang et al. proposed an empirical equation for the heat capacity estimation of two DESs^23^. H. Peyrovedin et al. proposed a correlation-based model aimed at estimating the speed of sound in 39 different deep eutectic solvents (DESs) across a broad temperature range. This model likely utilizes empirical correlations derived from experimental data to provide accurate predictions of sound speed in these solvents, which is essential for understanding their thermophysical properties and potential applications in various fields^24^. Their results indicated that the AARD% of the model is about 5.4%. Lapeña et al. correlated the speed of sound, heat capacity, density, isentropic compressibility, and viscosity of two DESs using a linear correlation^25^. Peng and Minceva utilized the perturbed chain polar statistical associating fluid theory (PCP-SAFT) model to predict the density and viscosity of DESs^26^. Lashkarbolooki et al. utilized the ANN to predict the heat capacity of binary ionic liquids mixtures^27^. They collected 1571 binary heat capacity data points for ILs. A neural network with one hidden layer containing 16 neurons successfully predicted IL binary heat capacities^27^. Thermodynamic models are a primary approach for predicting phase equilibrium and calculating second-order derivative thermodynamic properties

## Theory and methodology

### Data collection

As mentioned in the introduction section, the experimental data on the speed of sound are scarce.

In this study, 415 experimental data points of 38 DESs over a wide range of temperatures have been selected in the literature. The data points have been randomly divided into two sets containing 300 training and 115 testing data points. The most common train-test splits in the literature are 70:30 and 80:20, offering a good balance by providing enough data for both training and testing, and are often selected for their robustness and reliability in various contexts^44^. The training data points have been considered to develop the model, and the test data points have been utilized to check the model performance.

The input layer is critical in machine learning models. Predicting the speed of sound in DESs requires appropriate input features, likely drawn from various thermophysical properties. However, a challenge arises with newly introduced DESs, as their thermophysical properties are often unknown. This presents a problem for training accurate machine learning models to predict the speed of sound in these novel solvents. The group contribution methods can be utilized to estimate the thermo-physical properties of DESs such as critical pressure, critical temperature and critical volume, and acentric factor. In this work, the modified Lydersen and Joback–Reid GC method^45,46^ is used to estimate the thermo-physical properties of DESs. In Table 1 the groups parameters of the Lydersen and Joback–Reid method have been presented.

**Table 1:** Groups considered in the Joback–Reid method.

Group		${tb}_{k}$	${tc}_{k}$	${pc}_{k}$	${vc}_{k}$
Without rings					
–CH3		23.58	0.0141	− 0.0012	65
–CH2–		22.288	0.0189	0.0000	56
> CH–		21.74	0.0164	0.0020	41
> C <		18.25	0.0067	0.0043	27
=CH2		18.18	0.0113	− 0.0028	56
=CH–		24.96	0.0129	− 0.0006	46
=C <		24.14	0.0117	0.0011	38
–O–	[–O]^−^	22.42	0.0168	0.0015	18
> C=O		94.97	0.0284	0.0028	55
> N–	[> N <]^+^	11.74	0.0169	0.0074	9
–CN		125.66	0.0496	− 0.0101	91
–F	[F]^−^	− 0.03	0.0111	− 0.0057	27
–Cl	[Cl]^−^	38.13	0.0105	− 0.0049	58
–Br	[Br]^−^	66.86	0.0133	0.0057	71
–I	[I]^−^	93.84	0.0068	− 0.0034	97
–OH		92.88	0.0741	0.0112	28
With ring					
–CH2–		27.15	0.0100	0.0025	48
> CH–		21.78	0.0122	0.0004	38
> C <		21.32	0.0042	0.0061	27

Valderrama et al. estimated the critical points, normal boiling temperature, and acentric factor of ILs using the Joback method^45^. As shown in Table 1, the ion parameters have been considered based on the Valderrama et al. method. The normal boiling temperature (T_b_), critical temperature (T_c_), critical pressure (P_c_), critical volume (V_c_), and acentric factor (ω) are estimated using Eqs. (1)–(5) as follows:

1 $$T_{b} \left( K \right) = 198 + \mathop \sum \limits_{k} N_{k} tb_{k}$$

2 $$T_{c} \left( K \right) = T_{b} \left[ {0.584 + 0.965\left\{ {\mathop \sum \limits_{k} N_{k} tc_{k} } \right\} - \left\{ {\mathop \sum \limits_{k} N_{k} tb_{k} } \right\}^{2} } \right]^{ - 1}$$

3 $$P_{c} \left( {bar} \right) = \left[ {0.113 + 0.0032N_{atoms} - \mathop \sum \limits_{k} N_{k} pc_{k} } \right]^{ - 2}$$

4 $$V_{c} \left( {\frac{{cm^{3} }}{mol}} \right) = 17.5 + \mathop \sum \limits_{k} N_{k} vc_{k}$$

where *N*_*atoms*_ is the total number of atoms in the molecule. The acentric factors of ILs were estimated as follows^46^:

5 $$\omega = \frac{{\left( {T_{b} - 43} \right)\left( {T_{c} - 43} \right)}}{{\left( {T_{c} - T_{b} } \right)\left( {0.7T_{c} - 43} \right)}}\log \left( {\frac{{P_{c} }}{{P_{b} }}} \right) - \left( {\frac{{T_{c} - 43}}{{T_{c} - T_{b} }}} \right)\log \left( {\frac{{P_{c} }}{{P_{b} }}} \right) + \log \left( {\frac{{P_{c} }}{{P_{b} }}} \right) - 1$$

In Table 2 critical properties and acentric factor of DESs have been reported.

**Table 2:** The list of studied DESs and their thermo-physical properties that estimated by the modified Lydersen and Joback–Reid^45,46^, and references of speed of sound experiential data.

DESs	HBA	HBD	HBA:HBD molar ratio	Tc (K)	Pc (bar)	Vc (cm3/mol)	ω	Exp. data
DES1	1-Ethyl-3-methylimidazolium	Ethylene glycol	2:1	670.98	36.65	355.99	0.666	^47^
DES2	1-Ethyl-3-methylimidazolium	Ethylene glycol	1:1	651.23	39.77	308.96	0.7476	
DES3	1-Ethyl-3-methylimidazolium	Ethylene glycol	1:2	632.35	43.77	264.25	0.8293	
DES4	Benzyl-tributyl-ammonium-chloride	Ethylene glycol	1:3	657.28	31.24	364.48	0.9659	^48^
DES5	Benzyl-tributyl-ammonium-chloride	Diethylene glycol	1:3	720.58	25.62	480.62	0.9994	
DES6	Benzyl-tributyl-ammonium-chloride	Triethylene glycol	1:3	778.21	22.07	589.83	1.0507	
DES7	Benzyl-tributyl-ammonium-chloride	Glycerol	1:3	749.11	25.67	433.69	1.3146	
DES8	Benzyl-trimethyl-ammonium-chloride	Ethylene glycol	1:3	618.43	41.08	270.56	0.8745	
DES9	Benzyl-trimethyl-ammonium-chloride	Diethylene glycol	1:3	678.15	31.88	377.22	0.908	
DES10	Benzyl-trimethyl-ammonium-chloride	Triethylene glycol	1:3	733.31	26.6	478.88	0.9593	
DES11	Benzyl-trimethyl-ammonium-chloride	Glycerol	1:3	708.07	32.89	333.89	1.2232	
DES12	Benzyl-tripropyl-ammonium-chloride	Phenol	1:3	701.16	37.82	380.25	0.5152	^49^
DES13	Benzyl-tripropyl-ammonium-chloride	Ethylene glycol	1:3	644.1	33.78	334.18	0.9375	
DES14	Benzyl-tripropyl-ammonium-chloride	Lactic acid	1:3	721.27	33.15	384.56	0.9166	
DES15	Betaine	Glycerol	1:3	735.27	27.58	401.61	1.2862	
DES16	Betaine	Lactic acid	1:2	668.5	44.09	281.96	0.7863	^50^
DES17	Betaine	Lactic acid	1:5	683.07	47.23	259.82	0.8755	
DES18	Betaine	Levulinic acid	1:2	701.24	38.94	356.12	0.6195	
DES19	Betaine	Lactic acid/water	1:1:1	637.98	61.84	206.94	0.5794	
DES20	Choline-Chloride	Citric acid/water	2:1:6	659.71	92.43	146.46	0.5139	
DES21	Choline-Chloride	Urea	1:2	644.44	49.54	254.37	0.6509	^51^
DES22	Choline-Chloride	Ethylene glycol	1:2	602	40.99	259.67	0.9155	
DES23	Choline-Chloride	Glycerol	1:2	680.67	33.46	315.17	1.2254	
DES24	Choline-Chloride	Fructose	2:1	742.22	27.03	424.87	1.2278	
DES25	Choline-Chloride	Glucose	2:1	738.99	27.23	422.14	1.2163	
DES26	Choline-Chloride	1,2 propanediol	1:3	620.93	38.44	284.11	0.929	^52^
DES27	Choline-Chloride	Levulinic acid	1:2	702.19	35.4	376.78	0.7301	^53^
DES28	Choline-Chloride	Malonic acid	1:1	689.82	37.16	335.84	0.8577	^54^
DES29	Choline-Chloride	Glutaric acid	1:1	713.43	32.24	397.17	0.8782	
DES30	Choline-Chloride	Oxalic acid	1:1	676.24	40.44	303.06	0.8531	^55^
DES31	Dodecanoic	Octanoic acid	1:3	737.07	24.71	559.27	0.7649	^56^
DES32	Dodecanoic	Decanoic acid	1:2	773.88	21.55	656.4	0.8307	
DES33	Menthol	Octanoic acid	1:1	717.72	28.79	493.39	0.6173	
DES34	Menthol	Decanoic acid	1:1	739.17	26.26	549.11	0.6568	
DES35	Menthol	Salicylic acid	4:1	744.23	33.56	445.77	0.5733	^57^
DES36	Menthol	Ethylene glycol	1:1	654.33	38.54	319.91	0.751	
DES37	Proline	Levulinic acid	1:2	745.61	42.88	333.41	0.7044	^50^
DES38	Proline	Lactic acid	1:1	721.95	48.54	272.6	0.8243	

The GC method serves as an effective approach to estimate the thermophysical properties of DESs based on their molecular structures. This is particularly useful when experimental data is lacking, allowing researchers to generate necessary property estimates that can inform the modeling processes. In this work, the GC approach has been integrated with ANNs to predict the speed of sound in DESs.

Thoroughly analyzing and preparing input data is vital for building robust machine learning models. Each of these steps contributes to improving the quality of the input data, which in turn enhances the model’s ability to learn and generalize from the training data. The input data have been analyzed and reported in Table 3^58^.

**Table 3:** Statistical description of the data set used for modeling.

Parameters	U(m/s)	Tc (K)	Vc (cm3/mol)	w (−)	MW(g/mol)
Minimum	1166	602	146.46	0.5139	59.39
Maximum	2138	778.21	656.4	1.3146	190.61
Range	972	176.21	509.94	0.8007	131.22
Mean	1666.1	694.6	368.77	0.8625	125.00
Median	1688.5	701.2	356.055	0.8554	121.84
Standard deviation	208.99	45.35	108.34	0.216	30.289
Variance	43,679.34	2056.84	11,739.3	0.0468	917.46
Mid range	1652	690.105	401.43	0.9142	125
Interquartile range	244.77	77.99	140.76	0.2549	50.03
Sum of squares	11,880,780.5	76,103.2	434,351.36	1.734	34,863.54
Mean absolute deviation	160.65	38.55	83.28	0.1672	24.76
Root mean square	1679.11	696.04	383.95	0.888	128.53
Skewness	− 0.7036	− 0.164	0.643	0.483	0.1129
Kurtosis	2.965	2.393	3.751	2.893	2.767
Coefficient of variation	0.1254	0.0652	0.2938	0.251	0.2423
Relative standard deviation	12.54%	6.529%	29..38%	25.1%	24.23%

In the next section, the ANN + GC method has been presented.

### ANN methodology

**Fig. 1:**
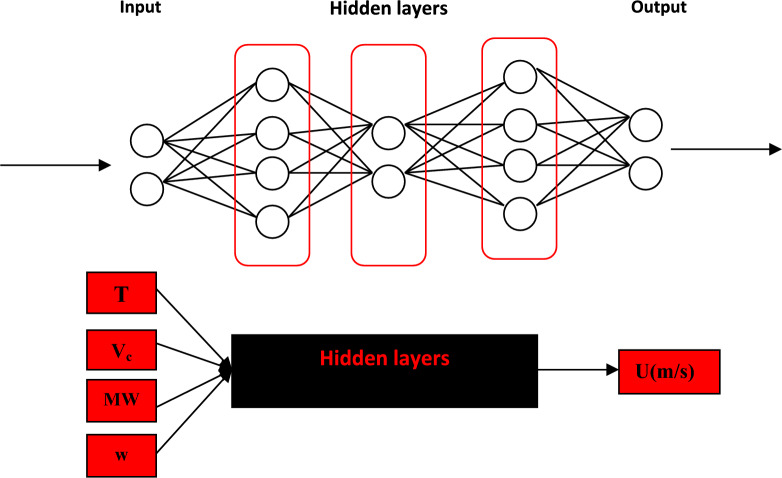
The neural network diagram.

The ANN can be likened to a black box with multiple inputs and outputs. The number of neurons can vary widely, ranging from fewer than ten to tens of thousands. These neurons can be organized into one, two, three, or more layers, with the two-layer network being the most commonly used ANN design for chemical applications. In the ANN methodology, inputs are fed into the input layer and then transmitted to the hidden layer using a specific transfer function. The transformed inputs are subsequently relayed to the output layer to estimate the desired properties. The estimated values are then compared to experimental data to analyze the error using the objective function (OF). Finally, the results are fed back into the system, and this process is repeated using a trial-and-error approach to minimize the objective function (OF) error. The number of neurons in the hidden layer is adjusted during these trials to achieve the best possible outputs with the lowest OF. This adjustment is guided by error analysis. Initially, one neuron is used to estimate the error with the training subset. Next, two neurons are evaluated, and this process continues, incrementing the number of neurons until the optimal configuration is found based on the minimum error value obtained from the testing subsets. Consequently, if the desired error is not achieved, the number of neurons in the hidden layer is increased. It must be noted that all input data are divided into two subsets namely training and testing. In Fig. 1 the schematic diagram of the neural network has been shown.

It must be noted that the weight parameters between neurons in hidden layers are utilized to develop the network. Summations of outputs of the previous layer in hidden layers must be weighted and added with bias. Hyperbolic tangent sigmoid transfer function is utilized in the hidden layer as follows:

6 $$n_{j} = \frac{2}{{1 + {\text{exp}}\left( { - 2Z} \right)}} - 1$$

7 $$Z = \mathop \sum \limits_{i = 1}^{r} w_{ij} p_{i} + b_{j}$$

where, $n_{j}$ is the *j*^th^ neuron output, $w_{ij}$ refers to weights of *i*^th^ neuron in the previous layer to the *j*^th^ neuron, $b_{j}$ is the bias, and $p_{i}$ is output. In this work, the weight and Bias of output and hidden layer have been reported in Table 3. The speed of sound of new-designed DESs can be predicted using the weight, Bias, and input values (obtained by the GC approach). The results of the ANN + GC model have been described in section “ANN+GC method”.

### Machine learning model

Machine learning is a subset of artificial intelligence (AI) that focuses on the development of algorithms and statistical models that enable computers to perform tasks without explicit instructions. Instead of being programmed with specific rules, machines learn from data and improve their performance over time. In this study the Categorical Boosting (CatBoost) machine learning approach has been used^59,60^. It excels at handling categorical features and often achieves state-of-the-art results in various machine learning tasks, particularly those involving tabular data. It’s designed to be easy to use, robust, and provides excellent accuracy. CatBoost directly handles categorical features without needing extensive preprocessing like one-hot encoding (though it can still benefit from good feature engineering)^61^. It uses a special method to encode categorical features called Target Statistics (also known as Ordered Target Encoding). This method reduces overfitting compared to naive target encoding. For each categorical feature, CatBoost calculates the average value of the target variable for each category. However, to prevent overfitting (a common problem with target encoding), it uses a more sophisticated approach to calculate these statistics. It avoids using the target value of the current row when calculating the target statistic for that row. This is done by calculating the target statistic based on the rows that came before the current row in the dataset’s order. Random permutations of the data are used to further reduce overfitting. CatBoost implements a variation of gradient boosting known as ordered boosting. This helps to address gradient bias that can occur in traditional gradient boosting algorithms, especially when dealing with categorical features. Gradient bias arises because the model is trained using the same data that was used to calculate the gradients, leading to an overestimation of the model’s performance. Ordered boosting aims to correct this bias. Like other gradient boosting methods, CatBoost iteratively builds an ensemble of decision trees. Each tree is trained to correct the errors made by the previous trees. The optimization process is driven by gradients calculated from a loss function. Within CatBoost, categorical boosting entails the utilization of categorical columns, incorporating permutation techniques such as one hot max size (OHMS) and target-based statistics. This method employs a greedy approach for each new split of the current tree, enabling CatBoost to unveil the exponential expansion of feature combinations^61^. The CatBoost method follows these steps:

- Formation of a random subset of the records
- Converting labels to integers
- Transforming categorical features into numeric values, as outlined below:
  8 $${\text{Average}}\,{\text{Target}} = \frac{{{\text{Count in Class }} + {\text{ Prior}}}}{{{\text{Total Count }} + { }1}}$$

**Fig. 2:**
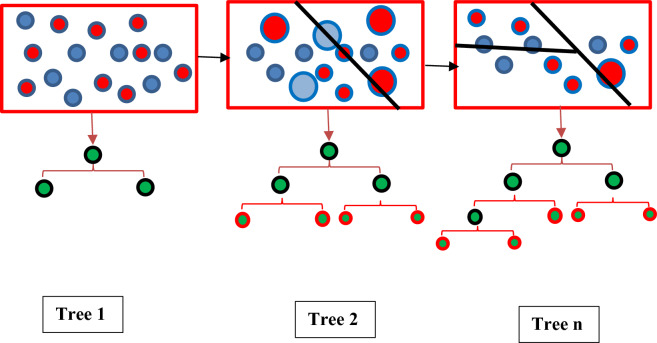
Schematic of the CatBoost tree construction.

In the equation provided, the parameter “Count in Class” aggregates the targets. Furthermore, each target is assigned a value of one and linked to specific categorical features, while the term “Total Count” tallies all previous instances^62^. In Fig. 2, schematic of the CatBoost tree construction has been depicted.

### Statistical error analysis

Statistical error analysis involves examining the errors and uncertainties in statistical measurements and data analysis. This is a crucial process in many fields, including science, engineering, economics, and social sciences, as it helps in assessing the reliability and validity of conclusions drawn from data. In this work the model results have been evaluated using the average relative deviation percent (ARD%), standard deviation (SD), mean absolute error (MAE), root mean square deviation (RMSE), and R^2^ values as follows:

9 $${\text{ARD}}\left( \% \right) = \frac{100}{N}\mathop \sum \limits_{i = 1}^{N} \left| {\frac{{U_{i}^{exp} - U_{i}^{calc} }}{{U_{i}^{exp} }}} \right|$$

10 $${\text{RMSE}} = \sqrt {\frac{{\mathop \sum \nolimits_{i = 1}^{N} (U_{i}^{exp} - U_{i}^{calc} )^{2} }}{N}}$$

11 $${\text{SD}} = \sqrt {\frac{{\mathop \sum \nolimits_{i = 1}^{N} \frac{{(U_{i}^{exp} - U_{i}^{calc} )^{2} }}{{U_{i}^{exp} }}}}{N - 1}}$$

12 $${\text{MAE}} = \frac{1}{N}\mathop \sum \limits_{i = 1}^{N} \left| {U_{i}^{exp} - U_{i}^{calc} } \right|$$

13 $$R^{2} = 1 - \frac{{\mathop \sum \nolimits_{i = 1}^{N} \left( {U_{i}^{exp} - \overline{U}_{i}^{calc} } \right)^{2} }}{{\mathop \sum \nolimits_{i = 1}^{N} (U_{i}^{exp} - \overline{U}^{exp} )^{2} }}$$

where $U_{i}^{exp}$ and $U_{i}^{calc}$ are experimental and estimated speed of sound of DESs. $\overline{U}^{exp}$ is the average value of the experimental speed of sound. In the next section the results of ANN + GC and ML + GC approaches have been presented.

## Results and discussion

In the previous section the ANN and ML methodologies for the prediction of the speed of sound of DESs has been described and depicted in Figs. 1 and 2. In this section the ANN + GC and ML + GC approaches have been described separately.

### ANN + GC method

The number of independent variables (input layer) plays a crucial role in ANN and ML methods^63^. In Table 1, thermodynamic properties of DESs containing T_c_, V_c_, MW, and ω have been reported. In the case of ANN method, different input properties and different numbers of neurons in one and two hidden layers have been considered, because one hidden layer only does not lead to adequate results^64–67^. The number of the hidden layer and neuron in the hidden layer and output are obtained using a trial and error algorithm. In this work the Levenberg–Marquardt algorithm^68,69^ is used to optimize the aforementioned parameters. The results show that, one hidden layer containing 16 neurons and four input properties containing the critical volume (V_c_), molecular weight (Mw), temperature, and acentric factor (ω) are the optimum values. As described in section “Theory and methodology”, 300 training and 115 testing data points of the speed of sound have been considered.

As mentioned in Eq. (7), the weight parameters between neurons in hidden layers are essential to develop the network. In Table 4 the weight parameters of neurons have been reported.

**Table 4:** The weight of hidden and output layers for 16 neurons.

Neurons	Hidden layer				Output layer
	V_c_	w	Mw	T	
1	− 2.24536	− 0.48558	1.098708	− 1.16422	0.630794
2	0.944691	− 2.52083	− 0.67325	0.373787	0.758028
3	− 1.01834	− 1.29702	1.427834	− 1.75557	0.977823
4	− 1.74537	1.232633	1.03008	− 1.4877	− 0.99896
5	1.276081	− 0.35078	− 1.66558	− 1.82055	0.730877
6	− 1.16913	1.762558	1.645331	0.812039	0.225133
7	− 0.31927	− 0.60319	2.715422	− 0.02673	0.9799
8	1.163699	1.667596	0.089376	− 1.92274	0.05536
9	− 0.93382	− 0.68464	2.549148	− 0.03313	− 0.04095
10	1.244022	1.623924	0.463342	− 1.85488	0.602695
11	− 0.47005	1.138715	− 1.54125	− 1.98668	− 0.54431
12	1.003801	− 0.67099	− 1.64259	1.919385	− 0.00381
13	1.557248	− 2.16729	− 0.7101	0.462178	0.801705
14	− 0.27949	1.406818	1.204804	2.081152	0.149322
15	− 1.77034	1.655671	1.199766	0.724712	0.690356
16	− 1.22579	− 1.24965	2.101388	0.599993	0.477281

**Fig. 3:**
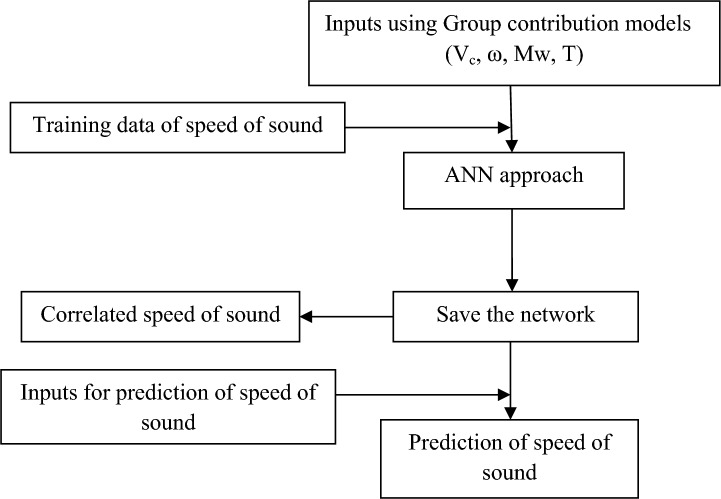
Flowchart of proposed model.

The optimum values of neurons in the hidden layer are evaluated using the average relative deviation percent (ARD%). When the optimum network architecture was determined, the input data of the ten DESs were fed to the network to predict their speed of sound. In Fig. 3 the flowchart of the proposed ANN model has been depicted.

**Fig. 4:**
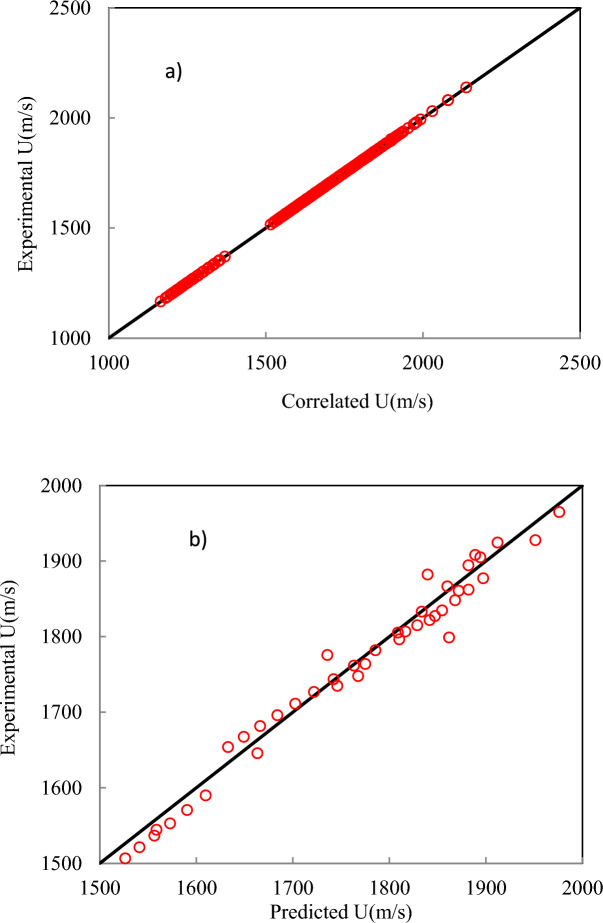
The results for the correlated (**a**) and predicted (**b**) speed of sound using the ANN + GC approach.

As shown in Fig. 3, the model can predict the speed of sound of DESs using independent variables containing T, V_c_, ω, and MW. The inputs containing V_c_, and ω can be estimated using GC approaches^45,46^. Experimental literature data is used as the training dataset for sound speed. An ANN with one hidden layer containing sixteen neurons is employed to develop the model*.* Four input variables can be fed into the saved file to generate predicted sound speed. Using the “saved network” and these four inputs, the sound speed of DESs can be accurately predicted. The complete MATLAB code, including all source files used in the programming, is provided in the Supplementary Material. The correlated and predicted results of the ANN + GC approach have been shown in Fig. 4.

**Fig. 5:**
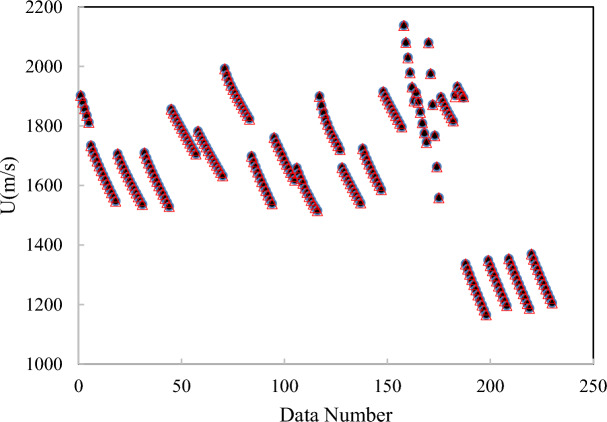
The speed of sound of DESs obtained using the ANN + GC. (●) Experimental data and (∆) ANN + GC.

Figure 4 shows that the ANN + GC approach can correlate the speed of sound of DESs over a wide range of temperatures, satisfactory. The ARD% and R^2^ of the correlated speed of sound have been obtained 0.032% and 0.9988, respectively. Figure 4b shows the prediction of the speed of sound of ten DESs using the ANN + GC approach. The results are in good agreement with experimental data. Figure 5 shows a simultaneous comparison between the experimental data and ANN + GC data.

**Fig. 6:**
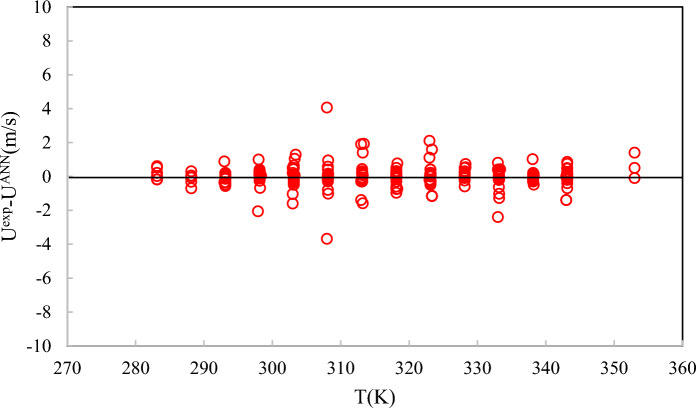
Deviations between calculations from ANN + GC and experimental speed of sound data at different temperatures.

As shown in Fig. 5, the ANN + GC method can correlate the experimental data accurately. Distributions of the deviation points for the ANN + GC method are shown in Fig. 6.

As shown in Fig. 6, the deviations between the ANN + GC predictions and experimental data do not exceed 4 m/s. Error analysis indicates that the proposed network is suitable for engineering calculations. In this study, the predictive performance of the ANN + GC model was assessed using R^2^, ARD%, SD, MAE, and RMSE metrics; see Table 5.

**Table 5:** Statistical error analysis for the ANN + GC model.

Model		ARD (%)	MAE	SD	RMSE	R^2^
ANN + GC	Train	0.024	1.5625	0.0538	2.1754	0.9981
	Test	0.053	1.4350	0.0524	2.2121	0.9990
	Total	0.032	1.5656	0.0549	2.2270	0.9988

As shown in Table 5, the total ARD%, MAE, SD, RMSE, and R^2^ values have been obtained 0.032%, 1.5656, 0.0549, 2.227, and 0.9988, respectively. The results of the ANN + GC approach show good agreement with experimental data. Models with high R^2^ values nearing 1 and low values for ARD%, RMSE, MAE, and SD are considered more accurate in predicting the speed of sound. In this study, the ARD% for the training and testing phases of the ANN + GC model were 0.024% and 0.053%, respectively. The overall R^2^ value approached unity at 0.9988. These results indicate that the ANN + GC model can accurately correlate the speed of sound in DESs across a wide temperature range. In the next section, the ML + GC model has been studied.

### ML + GC method

Similar to the ANN + GC method, the inputs for the ML + GC model included V_c_, ω, Mw, and T. Additionally, 300 training and 115 testing data points of the speed of sound were used to develop the machine learning approach. The statistical metrics for the CatBoost model are summarized in Table 6, which presents evaluations on the training and testing subsets (300 and 115 data points, respectively), as well as the complete dataset consisting of 415 points. In this study, the predictive performance of the models was assessed using R^2^, ARD%, SD, MAE, and RMSE metrics. Comparing model predictions with experimental data across both training and testing datasets provides valuable insights into the models’ accuracy and generalization capability; see Table 6.

**Table 6:** Statistical error analysis for the ML + GC model using CatBoot approach.

Model		ARD (%)	MAE	SD	RMSE	R^2^
CatBoost	Train	0.0381	0.9523	0.0516	2.1677	0.9991
	Test	0.0590	1.2349	0.0713	3.0208	0.9993
	Total	0.0332	0.9695	0.0556	2.3499	0.9992

**Fig. 7:**
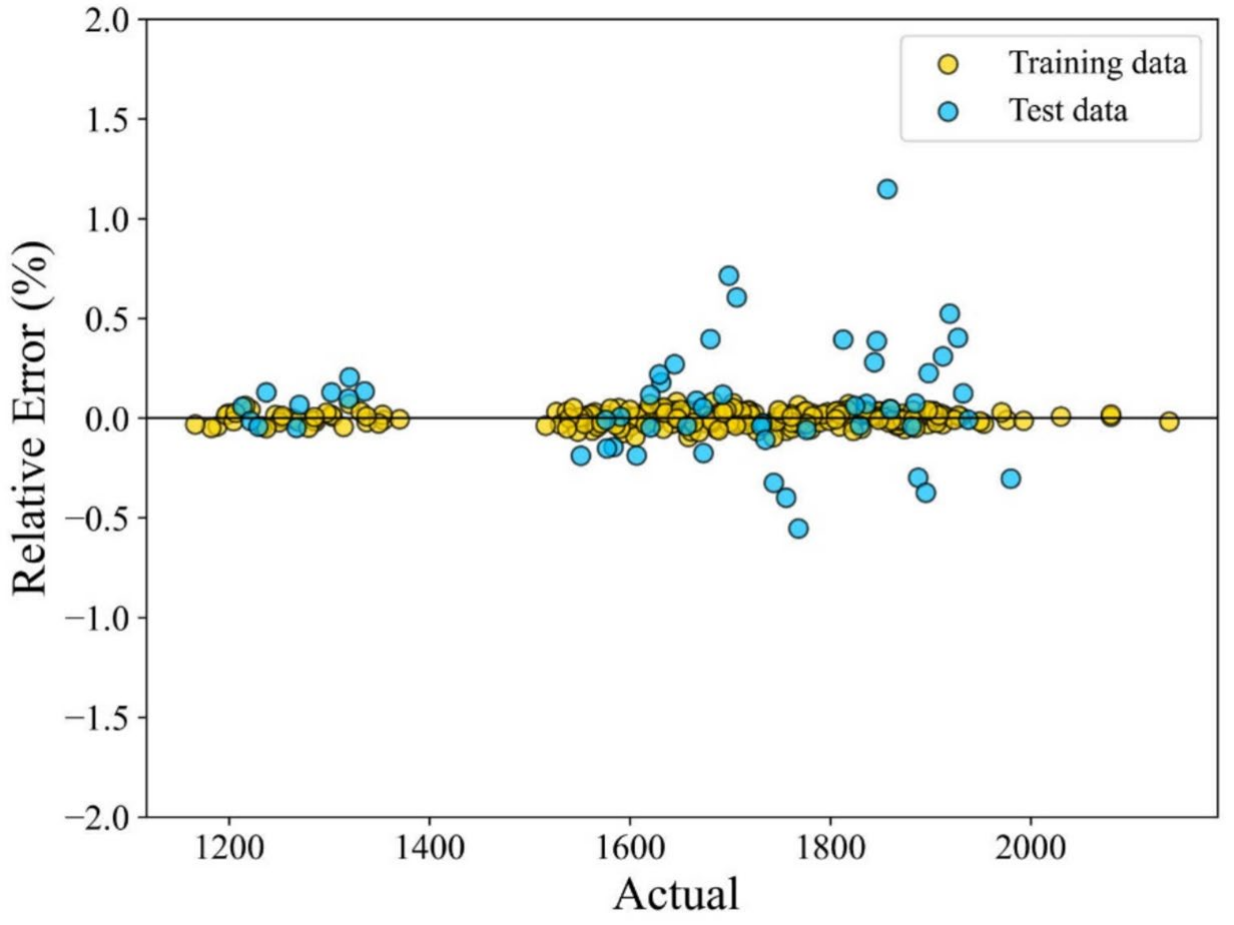
Error distribution plot of the ML + GC model to predict speed of sound.

**Fig. 8:**
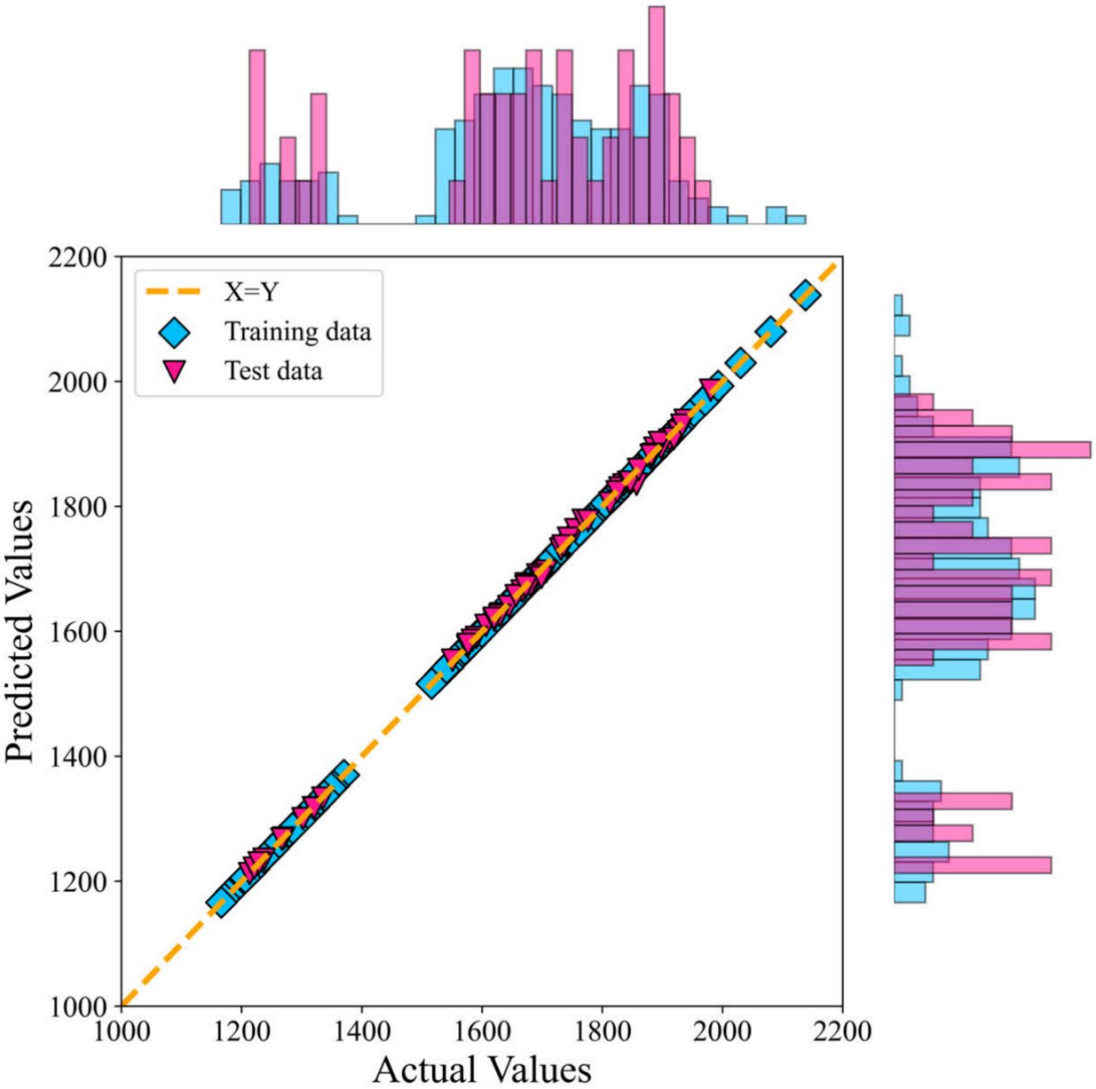
Cross-plot of the ML + GC model to predict speed of sound.

The greater the alignment between the predicted value and the experimental data, the higher the accuracy of the predictive model. In Figs. 7 and 8, the error distribution plot of the presented model vs the predicted speed of sound has been depicted. This visual representation demonstrates the robust agreement between the experimental data and the forecasts produced by the CatBoost ML method.

Figures 7 and 8 illustrate a strong correlation between the model-predicted data and the experimental data across both the training and testing datasets. These figures demonstrate a very close alignment between the model predictions and experimental points. In this research, graphical analysis complemented statistical methods to provide a more comprehensive evaluation of the models’ performance. These visual representations played a vital role in assessing the accuracy and reliability of the models. The percentage distribution of the relative error against the experimental values is presented in Fig. 7. In this type of error evaluation, relative error values are plotted against experimental output values. The closer the data points are to the zero-error line, the model is the more accurate. When the data points are scattered around the zero line, it indicates a significant difference between the predicted values and the experimental data, which proves the high error of the model. As a result, the proximity of the data points to the zero line for the ML + GC model indicates the high accuracy of this model. In Fig. 8, the cross-plot has been depicted. The cross-plot visually represents the comparison between predicted and experimental values. A closer alignment of data points with the unit slope line (Y = X) in the cross plot signifies higher accuracy and effectiveness of the model. The ML + GC model shows significant performance with most of the data points lying around the Y = X line. In the next section, a comparison between ANN + GC, ML + GC, and the correlation-based models has been investigated.

### Comparison between ANN + GC, ML + GC, and the correlation-based models

The ANN + GC and ML + GC results have been compared to five correlation-based models^24,70–73^. Singh and Singh proposed a correlation for speed of sound based on the surface tension and density^70^. Hekayati and Esmaeilzadeh suggested a novel interrelationship between surface tension (*σ*), density (*ρ*), and speed of sound (*u*) of ILs^71^. Gardas and Coutinho proposed a relationship between surface tension (*σ*), density (*ρ*), and speed of sound (*u*) for imidazolium based ILs, covering wide ranges of temperature, 278.15–343.15 K^73^. The aforementioned models are correlation-based. In Table 7 the ARD% of five correlation-based, ML + GC, and ANN + GC models have been reported and compared.

**Table 7:** ARD% values of ANN + GC, ML + GC, and five correlation-based models.

DESs	ML and ANN approaches			Correlations				
	Data set	ANN + GC	ML + GC	Peyrovedin et al. ^24^	Haghbakhsh et al.’s model ^72^	Hekayati and Esmaeilzadeh’s model ^71^	Gardas and Coutinho’s model ^73^	Singh and Singh’s model ^70^
DES1	Test	0.074	0.084	13.9	14.5	8.6	15.0	4.9
DES2	Train	0.008	0.020	7.5	16.6	3.6	9.0	11.8
DES3	Test	3.85	0.062	1.3	17.6	2.6	1.3	31.6
DES4	Train	0.012	0.105	5.8	2.4	8.1	5.2	33.0
DES5	Train	0.007	0.022	1.8	3.3	6.6	7.1	36.5
DES6	Train	0.006	0.047	0.9	8.3	5.0	9.3	43.8
DES7	Test	0.063	0.021	5.4	9.0	4.7	14.2	84.5
DES8	Train	0.006	0.043	0.7	68.8	2.8	1.9	27.2
DES9	Train	0.005	0.068	1.1	1.4	3.6	1.1	28.0
DES10	Test	0.042	0.042	2.8	7.0	2.1	2.9	32.1
DES11	Train	0.005	0.052	4.6	7.6	0.9	6.8	76.5
DES12	Train	0.023	0.189	1.9	2.7	3.6	3.6	4.4
DES13	Train	0.041	0.065	2.5	1.8	4.8	1.2	26.1
DES14	Train	0.055	0.036	8.9	22.0	11.9	13.7	54.0
DES15	Train	0.023	0.030	1.3	9.0	2.1	10.9	77.4
DES16	Train	0.012	0.124	1.6	1.3	4.6	2.8	37.4
DES17	Train	0.010	0.036	12.5	7.6	16.7	20.1	82.7
DES18	Train	0.003	0.059	2.8	2.0	4.9	1.0	15.8
DES19	Train	0.046	0.064	1.1	1.4	0.8	3.8	29.6
DES20	Test	0.092	0.053	2.4	1.5	1.8	6.2	79.5
DES21	Train	0.061	0.065	12.6	25.2	10.4	15.5	7.8
DES22	Test	0.030	0.026	4.7	10.4	2.6	7.9	13.8
DES23	Test	0.026	0.069	8.1	5.2	4.5	4.1	66.2
DES24	Train	0.029	0.032	1	4.7	4.6	3.6	55.2
DES25	Test	0.052	0.092	21.5	25.5	25.7	19.5	19.1
DES26	Train	0.007	0.054	2.5	11.6	8.8	4.0	36.6
DES27	Train	0.013	0.059	4.5	4.6	2.7	1.3	18.9
DES28	Train	0.104	0.029	5.9	3.6	3.7	4.2	30.9
DES29	Test	0.023	0.062	8.7	1.3	4.0	4.5	26.4
DES30	Train	0.018	0.105	3.9	6.8	2.6	2.7	39.3
DES31	Train	0.011	0.147	3.3	15.4	31.7	25.5	36.0
DES32	Train	0.017	0.0246	7.1	6.6	27.3	25.3	39.1
DES33	Train	0.018	0.216	7.7	13.1	29.0	19.5	22.7
DES34	Train	0.021	0.0459	2.1	11.6	26.1	18.9	23.5
DES35	Train	0.016	0.0313	9.2	3.1	24.3	17.8	36.8
DES36	Train	0.042	0.050	21.2	2.6	28.0	20.8	53.8
DES37	Test	0.05	0.075	6	2.2	12.8	13.7	56.8
DES38	Train	0.062	0.035	4.8	2.6	7.8	13.1	75.8
Average error	Test	0.053	0.059	–	–	–	–	–
	Train	0.024	0.038	–	–	–	–	–
	Correlated	–	–	5.67	9.52	9.38	9.45	38.83

**Fig. 9:**
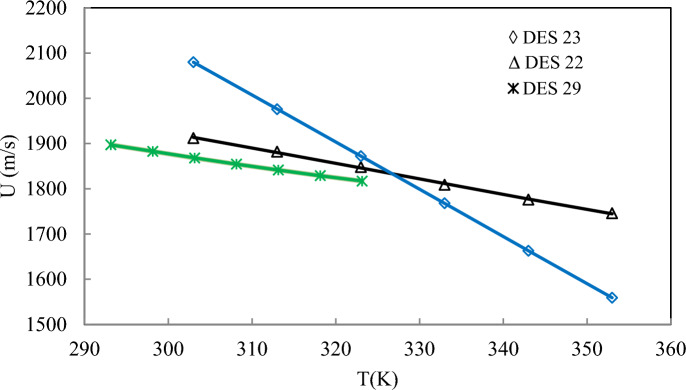
Prediction of speed of sound of DESs using ANN + GC approach. Lines are model prediction and symbols are experimental data. Standard uncertainty of DES speed of sound is 1.0 m/s.

The average ARD% value of Peyrovedin et al.^24^ model was obtained 5.67%. ARD% values of Haghbakhsh et al.’s model^72^, Hekayati and Esmaeilzadeh’s model^71^, and Gardas and Coutinho’s model^73^ for 38 DESs have been obtained 9.52%, 9.38%, and 9.45%, respectively. The average ARD% value of Singh and Singh’s model^70^ was obtained about 39%. They correlated the speed of sound of ILs using surface tension and density data. As shown in Table 7, the Peyrovedin et al.^24^ model gives a lower ARD% value compared to other correlation-based models. The ANN + GC and the ML + GC models give lower error values compared to correlation-based models. The ARD% of the ANN + GC model is slightly lower than the ML + GC model. In Fig. 9 the speed of sound of some DESs using the ANN + GC approach have been compared to experimental data.

**Fig. 10:**
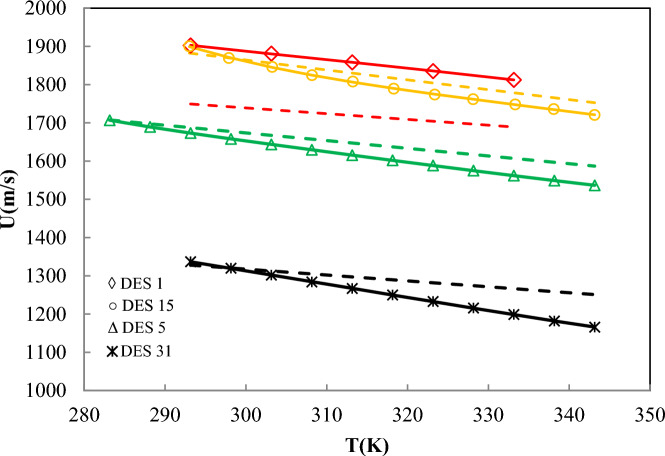
Correlation of speed of sound of DESs using ANN + GC approach (lines) and Peyrovedin et al. model (dashed-lines). Symbols are experimental data. Standard uncertainty of DES speed of sound is 1.0 m/s.

As shown in Fig. 9, the ANN + GC correlates the speed of sound of DESs satisfactory. The average ARD% of the ANN + GC model was obtained 0.032%. In Fig. 10, the ANN + GC model results have been compared to experimental data and H. Peyrovedin et al. model.

**Fig. 11:**
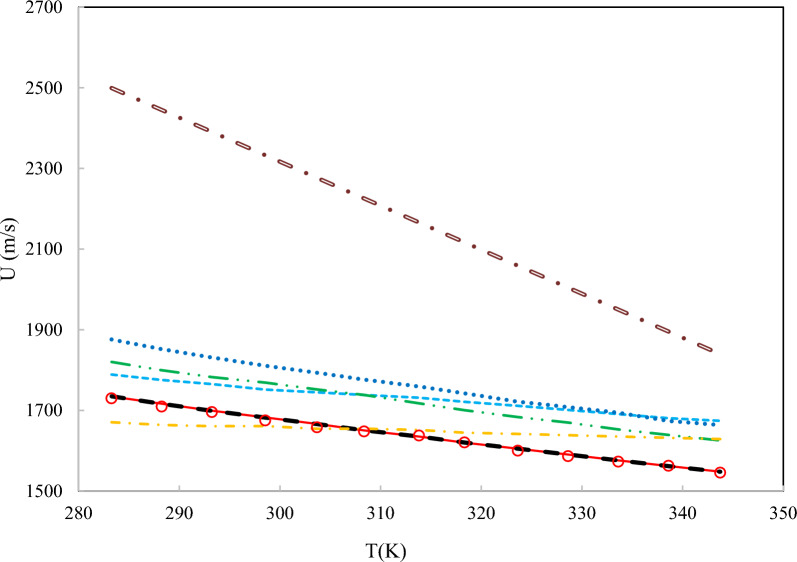
Comparison of the behavior of the speed of sound of DES 4 versus the temperature for the ANN + GC model, ML + GC model, and literature models. (-) ANN + GC, (--) ML + GC, (- - -) H. Peyrovedin et al.^24^, (-..-) Haghbakhsh et al.’s model^72^, (…) Hekayati and Esmaeilzadeh’s model ^71^, (-.-) Gardas and Coutinho’s model^73^, and (=.=) Singh and Singh’s model^70^. Symbols are experimental data.

As depicted in Fig. 10, the ANN + GC approach correlates the speed of sound of four DESs at various temperatures accurately. In the case of DES1, the ARD value of H. Peyrovedin et al. model is higher than ANN + GC, nevertheless, their obtained results are acceptable. Figure 10 shows that, their proposed correlation is accurate at lower temperatures, and the model deviations are increased by increasing temperature. As reported in Table 7, and Figs. 9 and 10, the average ARD% value of the testing and training results of the ANN + GC are acceptable. In Fig. 11, the ANN + GC model has been compared to the ML + GC and five correlation-based models.

**Fig. 12:**
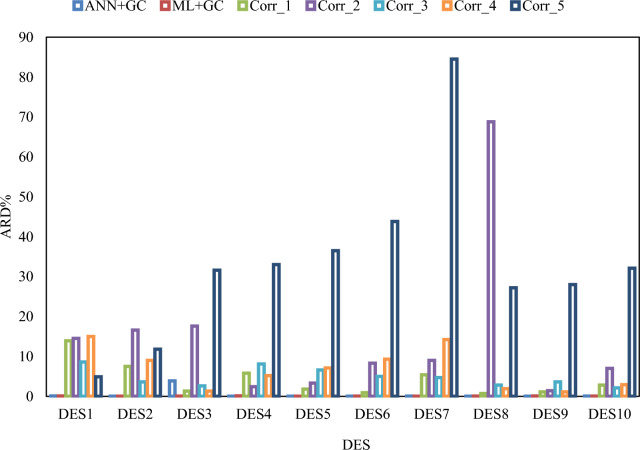
Error distribution plot for ten DESs. Corr_1, Corr_2, Corr_3, Corr_4, and Corr_5 refer to H. Peyrovedin et al.^24^, Haghbakhsh et al.’s model^72^, Hekayati and Esmaeilzadeh’s model^71^, Gardas and Coutinho’s model^73^, and Singh and Singh’s model^70^.

As shown in Fig. 11, the average ARD% values of the ANN + GC approach are lower than the correlation-based models. The average error values of ANN + GC and ML + GC models are comparable. In Fig. 12, the error distribution plot for ten DESs has been depicted.

**Fig. 13:**
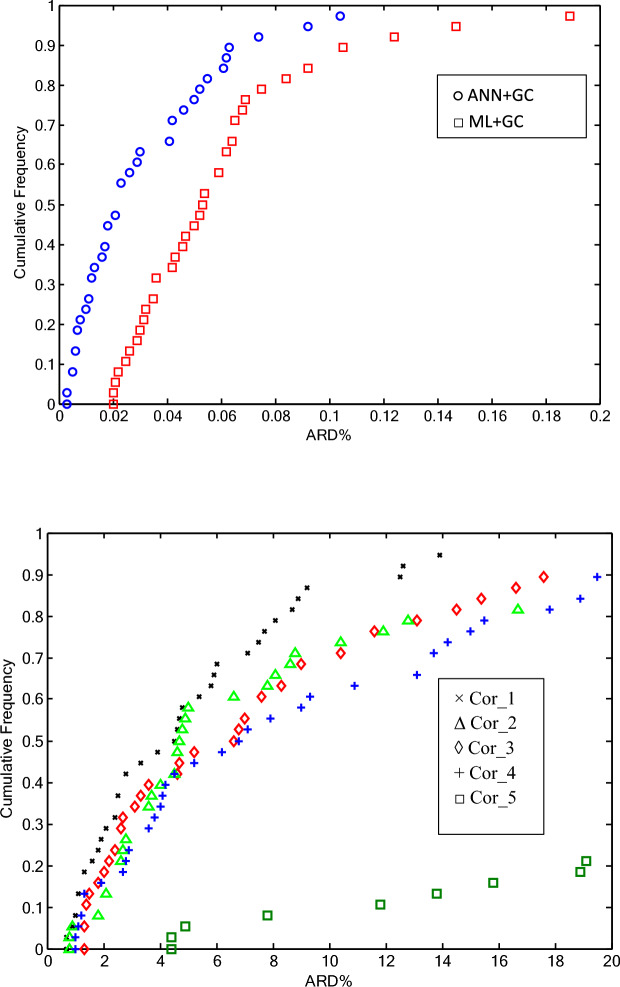
Cumulative frequency plot for all studied DESs. (**a**) ANN + GC and ML + GC methods, (**b**) Corr_1, Corr_2, Corr_3, Corr_4, and Corr_5 refer to H. Peyrovedin et al.^24^, Haghbakhsh et al.’s model^72^, Hekayati and Esmaeilzadeh’s model^71^, Gardas and Coutinho’s model^73^, and Singh and Singh’s model^70^, respectively.

Cumulative frequency diagrams are one of the graphical methods used for evaluating model performance^74^. Figure 13a and b illustrate the cumulative frequency diagrams of the ANN + GC and ML + GC models, along with five correlations (as reported in Table 7).

As shown in Fig. 13a, approximately 90% of the values estimated by the ANN + GC model exhibited an ARD% of less than 0.07%. In the case of ML + GC model, 90% of the ARD% values are less than 0.1%. In Fig. 13b, the cumulative frequency of five correlation-based models has been depicted. The correlation developed by Singh and Singh’s^70^ demonstrated poor performance. The results show that, the ANN + GC model achieves high precision in forecasting speed of sound of DESs compared to the five correlation-based model.

### The leverage approach for model analysis

The leverage approach is a valuable tool for ensuring the quality and reliability of statistical models. Identifying and addressing high-leverage points, can improve model accuracy, enhance data understanding, and lead to more informed decision-making^75^. Leverage values help identify observations that have a disproportionate influence on the regression coefficients. Points with high leverage and large residuals are particularly problematic, as they can significantly distort the model fit. Leverage diagnostics are used during model validation to assess the stability and generalizability of the model. If the model is highly sensitive to a few high-leverage points, it may not perform well on new data. High-leverage points often indicate data errors or unusual events. Identifying these points allows for a targeted investigation of the data to identify and correct errors or to understand the underlying causes of the unusual observations. High leverage points can sometimes indicate the need to include additional predictor variables in the model. In some cases, transforming the predictor or response variables can reduce the influence of high-leverage points and improve the model fit. As well, investigating high-leverage points can provide valuable insights into the data and the underlying processes that generated it. In this study the leverage approach has been utilized to study the ANN + GC model. In this regard, standardized residuals (SR) and Leverage values, derived from the diagonal elements of the hat matrix have been calculated. The hat matrix was given by:

14 $$H = X\left( {X^{t} X} \right)^{ - 1} X^{t}$$

where $X^{t}$ refers to the transpose of matrix X. The critical leverage was calculated as 3(n + 1)/m. where m and n represent the number of data points and model input variables, respectively. The applicability domain of the ANN + GC model can be assessed by plotting standardized residuals against leverage values (Williams plot). The Williams plot is the most common and direct way to do this. The applicability domain (AD) of a model is the region where the model is considered reliable for making predictions. In simpler terms, it’s the set of conditions under which you can trust the model’s output. Extrapolating beyond the AD can lead to inaccurate or unreliable predictions.

By plotting standardized residuals against leverage values against each other, the Williams plot allows you to identify observations that:

- Are outliers (large standardized residuals)
- Have high leverage (unusual predictor values)
- Are both outliers and have high leverage (potentially very influential and problematic)

**Fig. 14:**
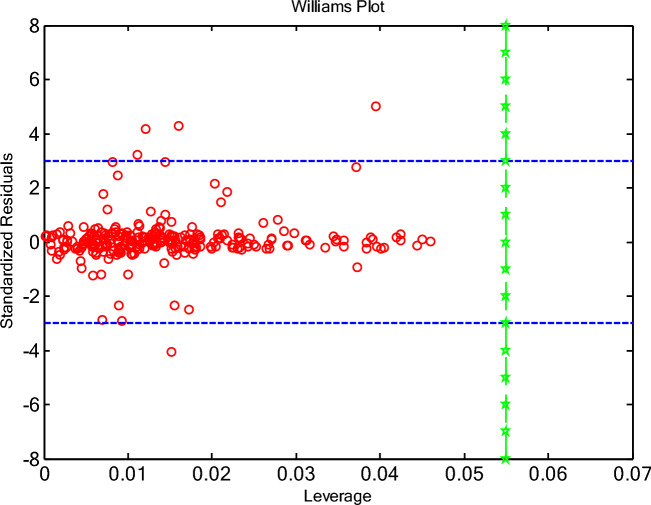
The Williams plots for outlier detection using the ANN + GC model.

If the majority of data points were situated within the boundaries of the 0 ≤ H ≤ critical leverage, and—3 ≤ SR ≤ 3, the established model is deemed reliable, and its predictions are confined within the applicability domain^75^. In Fig. 14, the William’s plot is illustrated.

As shown in Fig. 14, the critical leverage value has been obtained about 0.0545. As depicted in Fig. 14, most of the data point falls between 0 ≤ H ≤ 0.0545, and—3 ≤ SR ≤ 3. The results indicated that, the ANN + GC model is highly reliable. There are some suspicious data (SR > 3 or SR <—3). Figure 14 shows that, only five data points have an SR-value outside the range of—3 to 3, classifying them as questionable data. On the other hand, all data points have H values lower than 0.0545. This result indicated that all data points have satisfactory leverage. The Leverage approach confirms the accuracy of databank and the high reliability of ANN + GC model in estimating speed of sound of DESs.

In the next section, the sensitivity analysis (SA) of input variables in the ANN + GC model has been studied.

### Sensitivity analysis

**Fig. 15:**
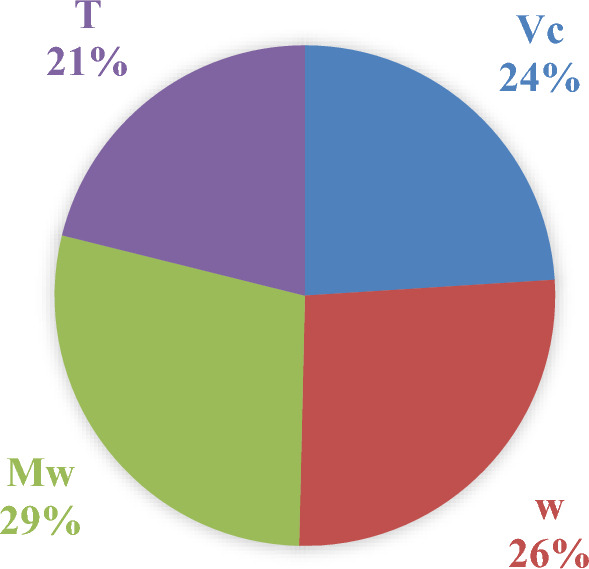
Relative importance (%) of input variables on the value of the speed of sound of DESs.

Sensitivity analysis in ANNs involves determining how much each input variable influences the network’s output. It helps you understand which inputs are most important and how changes in those inputs affect the model’s predictions. Sensitivity analysis using weight-based methods involves evaluating the influence of input variables on the output by analyzing the weights within the network. These methods are generally more straightforward and computationally less expensive than perturbation-based methods. Garson suggested an equation based on partitioning of connection weights for sensitivity analysis of input variables as follows^76^:

15 $$IF_{j} = \frac{{\mathop \sum \nolimits_{m = 1}^{Nh} \left( {\left( {\frac{{\left| {w_{jm}^{ih} } \right|}}{{\mathop \sum \nolimits_{k = 1}^{Ni} \left| {w_{km}^{ih} } \right|}}} \right).w_{mn}^{ho} } \right)}}{{\mathop \sum \nolimits_{k = 1}^{Nh} \left\{ {\mathop \sum \nolimits_{m = 1}^{Nh} \left( {\left( {\frac{{\left| {w_{km}^{ih} } \right|}}{{\mathop \sum \nolimits_{k = 1}^{Ni} \left| {w_{km}^{ih} } \right|}}} \right).w_{mn}^{ho} } \right)} \right\}}}$$

where *IF*_*j*_ is the relative importance of the *j*^*th*^ input variable on output variable; *N*_*i*_ and *N*_*h*_ refer to the number of input and hidden neurons, respectively. The superscripts *i*, *h* and *o* refer to input, hidden and output layers, respectively. The subscripts *k*, *m* and *n* refer to input, hidden and output layers, respectively. *w* is connection weights. The relative importance of input variables (IF_j_) were calculated by Eq. (15). This approach expands on Garson’s method by considering the direct and indirect paths from inputs to outputs. It involves calculating the influence of each input across the network layers into the final output. In Fig. 15 the importance of input variables based on normalized percentage has been depicted.

It is evident that all selected input variables have a strong influence on the speed of sound values, with importance levels ranging from 21 to 29%. However, it is important to note that highly nonlinear models or coupled input variables can complicate sensitivity analysis. These results highlight which inputs have the most significant impact on the output, aiding in model refinement, feature selection, or providing insight into the underlying process. As shown in Fig. 15, the contributions are typically normalized to sum to 1 (or 100%) to facilitate easier interpretation of the results.

In summary, ANN methods have several key advantages and disadvantages^77^. They can model complex nonlinear relationships by selecting an appropriate architecture through trial and error. Once the input layer, the number of neurons, and hidden layers are established, ANNs can predict values beyond those considered during training without reprogramming. However, acquiring large datasets is often challenging and time-consuming. Additionally, the complexity of ANNs can make their implementation difficult. Another drawback is that ANNs require robust central processing units (CPUs) or hardware, which can be resource-intensive. This study demonstrates the strong performance of ANN models in predicting second-order derivative thermodynamic properties, such as the speed of sound in DESs, despite the aforementioned limitations. Traditionally, equations of state (EoS) models have been widely used to estimate the thermo-physical properties of complex systems like ILs and DESs. However, predicting the speed of sound using EoS-based models requires the ideal gas heat capacity of the pure components. Estimating this property using GC models often results in significant deviations in some cases. Consequently, researchers are seeking alternative approaches to predict the speed of sound and specific heat capacity without relying on ideal gas heat capacity estimations. This work shows that the ANN + GC method can be considered a robust and efficient alternative, particularly for predicting second-order derivative thermodynamic properties, such as the speed of sound.

## Conclusions

In this work, the Group Contribution (GC) approach was employed to estimate the input variables for the ANN model. Critical properties and the acentric factor were determined based on the molecular structure of DESs, utilizing the Lydersen and Joback–Reid GC methods. The combined ANN + GC model was developed to predict the speed of sound in DESs across a wide temperature range. For model development, 415 data points from 38 DESs were selected. The results indicate that a single hidden layer with sixteen neurons provides optimal values for ARD% and R^2^. The model’s performance was evaluated using several metrics: average relative deviation percent (ARD%), standard deviation (SD), mean absolute error (MAE), root mean square deviation (RMSE), and R^2^. The findings demonstrate that the ANN + GC model can accurately estimate the speed of sound in DESs over a wide temperature range. The obtained values for the metrics were ARD% = 0.032%, SD = 0.0549, MAE = 1.5656, RMSE = 2.227, and R^2^ = 0.9988. The model results were compared to five correlation-based models and a Machine Learning (ML) model. Similar to the ANN + GC approach, the GC method was combined with the ML model. The results indicated that the ML + GC and ANN + GC models were comparable; however, the ANN + GC model showed slightly lower error values. Overall, the error metrics for the ANN + GC approach were lower than those of the five correlation-based models. The cumulative frequency diagrams and the leverage approach were implemented to validate the quality and reliability of the proposed model. The leverage analysis confirmed the accuracy of the data used and the high reliability of the ANN + GC model for estimating the speed of sound in DESs. The primary goal of this study was to predict the speed of sound in DESs based solely on their molecular structure, without relying on any experimental data. This work demonstrates that the ANN + GC method can serve as a robust model for predicting second-order derivative thermodynamic properties of DESs in the absence of experimental measurements. The proposed model could be employed in future studies, particularly in the pre-design of new solvents.

## Supplementary Information

Supplementary Information.

## Data Availability

All data generated or analysed during this study are included in this published article [and its supplementary information files].

## List of symbols

ARD (%): Average relative deviation
$$b_{j}$$: Bias
*IF*_*j*_: Relative importance of the *j*^*th*^ input variable on output variable
*i*: Input layers
*h*: Hidden layers
*k*: Input layers
*m*: Hidden layers
MAE: Mean average error
$n_{j}$: The *j*^th^ neuron output
*N*_*atoms*_: Total number of atoms
*N*_*i*_: Number of inputs
*N*_*h*_: Hidden neurons
*n*: Output layers
*o*: Output layers
$$p_{i}$$: Output
P_c_: Critical pressure
RMSE: Root Mean Square Deviation
R^2^: Coefficient of determination
SD: Standard deviation
T_b_: Normal boiling temperature
T_c_: Critical temperature
V_c_: Critical volume
$w_{ij}$: Weights of *i*^th^ neuron in the previous layer to the *j*^th^ neuron
*w*: Connection weights
ω: Acentric factor
