# Supplementary material for: Prediction of speed of sound of deep eutectic solvents using artificial neural network coupled with group contribution approach
Source: Sci Rep. 2025 Aug 10;15:29238. doi: 10.1038/s41598-025-14094-w (PMC12336353; doi:10.1038/s41598-025-14094-w)
Supplement: Supplementary file 1 — Supplementary Information. [file 41598_2025_14094_MOESM1_ESM.docx]

**Appendix A.**

%ANN for speed of sound of Deep eutectic solvents

%Training

p = xlsread('VAR_training_U');p=p'; % Read the dependent variables (Vc,T, w, Mw)

t = xlsread('U_for_training');t=t'; % Reading the dependent variable (U for training)

[pn,minp,maxp,tn,mint,maxt] = premnmx(p,t); % Normalization of all data (values between -1 y +1)

net=newff(minmax(pn),[5,32,1],{'tansig','tansig','purelin'},'trainlm');

net.trainParam.show = 10;

net.trainParam.epochs = 6000; net.trainParam.goal = 1e-7;

w1 = net.IW{1,1}; w2 = net.LW{2,1}; w3 = net.LW{3,2};

b1 = net.b{1}; b2 = net.b{2}; b3 = net.b{3};

before_training = sim(net,pn);

corrbefore_training= corrcoef(before_training,tn);

[net,tr]=train(net,pn,tn);

after_training = sim(net,pn);

after_training = postmnmx(after_training,mint,maxt); after_training=after_training';

Res = sim(net,pn);

dlmwrite('U_correlated.xls',after_training,char(9));

save w1_U

%Prediction section

load w1_ U

pnew = xlsread('VAR_U_ prediction'); pnew=pnew';

pnewn = tramnmx(pnew,minp,maxp);

anewn = sim(net,pnewn);

anew = postmnmx(anewn,mint,maxt); anew=anew';

dlmwrite('U_ prediction.xls',anew,char(9));
